# Informal Patient Payments and Bought and Brought Goods in the Western Balkans – A Scoping Review

**DOI:** 10.15171/ijhpm.2017.73

**Published:** 2017-07-03

**Authors:** Sofie Buch Mejsner, Leena Eklund Karlsson

**Affiliations:** Unit for Health Promotion Research, University of Southern Denmark, Esbjerg, Denmark.

**Keywords:** Scoping Review, Informal Patient Payments, Bough and Brought Goods, Health care, Western Balkan

## Abstract

**Introduction:** Informal patient payments for healthcare are common in the Western Balkans, negatively affecting public health and healthcare.

**Aim:** To identify literature from the Western Balkans on what is known about informal patient payments and bought and brought goods, to examine their effects on healthcare and to determine what actions can be taken to tackle these payments.

**Methods:** After conducting a scoping review that involved searching websites and databases and filtering with eligibility criteria and quality assessment tools, 24 relevant studies were revealed. The data were synthesized using a narrative approach that identified key concepts, types of evidence, and research gaps.

**Results:** The number of studies of informal patient payments increased between 2002 and 2015, but evidence regarding the issues of concern is scattered across various countries. Research has reported incidents of informal patient payments on a wide scale and has described various patterns and characteristics of these payments. Although these payments have typically been small – particularly to providers in common areas of specialized medicine – evidence regarding bought and brought goods remains limited, indicating that such practices are likely even more common, of greater magnitude and perhaps more problematic than informal patient payments. Only scant research has examined the measures that are used to tackle informal patient payments. The evidence indicates that legalizing informal patient payments, introducing performance-based payment systems, strengthening reporting, changing mentalities and involving the media and the European Union (EU) or religious organizations in anti-corruption campaigns are understood as some of the possible remedies that might help reduce informal patient payments.

**Conclusion:** Despite comprehensive evidence regarding informal patient payments, data remain scattered and contradictory, implying that informal patient payments are a complex phenomenon. Additionally, the data on bought and brought goods illustrate that not much is known about this matter. Although informal patient payments have been studied and described in several settings, there is still little research on the effectiveness of such strategies in the Western Balkans context.

## Introduction

Informal patient payments for healthcare are common in Central, Eastern and Southern European countries.^1-5^ In the early transition from communist countries, this region experienced a decline in economic production, reducing the capacity of government spending on health.^6^ In the aftermath of the break-up of Yugoslavia, most of the Western Balkan countries (Albania, Bosnia and Herzegovina, Croatia, Kosovo, Former Yugoslav Republic of Macedonia [FYROM], Montenegro and Serbia) introduced a social insurance system and increased out-of-pocket (OOP) payments to finance health care,^7^ facilitating both formal and informal patient payments. Such increase in private OOP spending, creates a wider network of providers and increases accessibility, but makes paying difficult for some vulnerable groups. On the contrary, publicly financed health care protects the population from financial risks by providing not for profit and affordable health care, that everyone is eligible to.^8^ Though such accessibility may be limited in terms of long waiting lists and eligibility criteria (citizenship, age etc).

Informal patient payments are conceptually part of the broader term ‘OOP payments,’ which is defined as “private health expenditure paid directly by individuals to health care providers when in contact with the health care system.”^8,9^ Countries with fewer resources for public health spending often experience growth in private (formal or informal) OOP payments, shifting the costs to the health consumers.^10^ It is likely that most private spending takes the place of informal patient payments,^11^ due to underfunded, ineffective and poorly functioning (public) health care systems. OOP payments vary among countries, but a strong relationship is observed between the level of OOP payments for health and the extent of catastrophic and impoverishing health expenditures in a country, generating problems of financial protection of the population.^8,10^

Informal patient payment is defined as “a direct contribution, which is made in addition to any contribution determined by the terms of entitlement, in cash or in-kind (gratitude gift), by patients or others acting on their behalf, to healthcare providers for services that the patients are entitled to.”^12^ The payments may be in the form of flowers, sweets, small tips or large sums of cash.^12^ By definition, informal patient payments also include bought and brought goods, although the two terms differ in practice because ‘bought and brought goods’ involve payment for goods (such as pharmaceuticals, bed linen, materials or equipment) that patients are requested to bring with them when seeking healthcare.^12,13^ Bought and brought goods are therefore not counter-favors but are brought by patients as part of the process of seeking required service delivery. A distinction should be made between goods (products) and services (assistance or advice) in health care, as different practices exist around paying for services or providing goods that one is entitled for. As it is now, the term ‘informal patient payments’ includes in kind gifts or bribery and bought and brought goods. This study separates these two terms because bought and brought goods are not as recognized as informal patient payments.^14^ The present study will therefore give a more nuanced and clearer distinction of the research available in this field.

OOP payments are important to examine due to the financial risk that these pose on vulnerable groups.^15^ Data from 6 of the Western Balkan countries (not including Kosovo) indicate that public health expenditure^ [1]^ accounts for approximately 50%-80%, where Albania has the lowest public health expenditure and Croatia the highest.^16^ OOP spending is therefore quite high in some Western Balkan countries, particularly in Albania and Montenegro. Prior research suggests that OOP payments – including informal patient payments and bought and brought goods – negatively affect public health, access to healthcare and health equity.^17,18^ These payments may further lead consumers to curtail their use of health services, affecting poorer social groups, in particular.^15^ Especially informal patient payments are necessary to study since patients often mistakenly perceive informal patient payments as official fees, and they may therefore be difficult to capture.^19^ Because of such payments, it has been difficult to precisely estimate expenditures for healthcare services in affected areas. In the seven Western Balkan countries, healthcare is publicly financed by the income tax system. Formal and private OOP payments are an additional source of funding in these health systems, although informal patient payments and bought and brought goods may inflate them further.^20^

The Euro Health Consumer Powerhouse^21^ ranked the performance of European health systems performance in 2015 using 48 indicators. Of 35 European countries, the Western Balkan healthcare systems ranked from lowest to intermediate levels of performance, with Montenegro at the bottom. Bosnia and Herzegovina was not included in this edition, due to an inability to furnish reliable data.^21^ One explanation for the poor performance in these countries may be found in Transparency International’s (TI)^22^ Corruption Perception Index, which ranked the countries comprising the Western Balkans from 64th to 110th out of 177 countries in terms of the level of corruption [an overall perception of corruption that included not just healthcare but all public sectors].

In most Western Balkan countries, anti-corruption laws prohibit providers from receiving informal patient payments, such as gifts and bribes (eg, in Albania and Serbia^23,24^). However, enforcement of these laws is weak and inefficient in these countries, resulting in minimal legislative impact on corruption.^25,26^ Even Croatia, the lone Western Balkan country in the European Union (EU), continues to struggle with bribes in the form of gifts. Although bribery is illegal, gifts are legal in Croatia based on their value and intent, which leads to the use of gifts as bribes.^27^

### Objectives of the Study

This study aims to answer the following research questions:

- For Western Balkan countries, what is known about informal patient payments and bought and brought goods and their effects on healthcare systems over the past 13 years?
- What actions can be taken to tackle this problem?

As there are no governmental regulations that specifically cover ‘bought and brought goods,’ the practices involving them typically do not contravene established laws or regulations. Further, this issue has not been tackled by policy-makers.^28^ However, according to living standard measurement surveys in Albania,^29^ for example, services and drugs that should be provided for free are often paid for by patients. The existence of informal patient payments thus warrants examination, particularly because Western Balkan countries are beginning to apply to and join the EU. As Ahrens^30^ argues, the existence of informal patient payments is a sign of poor governance that may hamper these countries’ transition into the EU. The present review is among the few that considers both bought and brought goods, informal patient payments and measures to tackle these payments and goods. Further, the review distinctively compares all 7 Western Balkan countries in terms of these three issues.

In the coming sections, a detailed methodological description is presented, together with an illustration of the identification of papers and a quality assessment of the findings.

## Methods

We conducted a scoping review to map the state of the literature in the area of concern. A scoping review, which has been previously described by Arksey and O’Malley,^31^ is a form of knowledge synthesis that addresses an exploratory research question and aims to map key concepts, types of evidence, and gaps in research that are related to a defined field.^32^ Levac et al,^33^ Daudt et al,^34^ and Colquhoun et al^32^ address the strengths and limitations of scoping review methodology and encourage consistency in methods across scoping studies. The present review is based on the scoping review framework developed by Levac et al^33^ and Daudt et al.^34^

### Search Strategy

The search process was iterative, and it continuously included and excluded studies and redefined search terms in the process of becoming familiar with the literature. Therefore, the steps of phrasing the research question, searching and selecting relevant published and grey literature was repeated to ensure the comprehensiveness of the evidence.

The main search terms that were used included “health systems,” “Western Balkan” and “informal payments.” These included a total of 99 sub-keywords connected with the Boolean operator OR. The sub-keywords were for example “health governance,” “healthcare system,” “health professional” or “Western Balkan” or individual countries, eg, “Serbia,” “Croatia.” Sub-keywords for “informal payments” were terms such as “informal payment,” “bribe,” “drug,” “goods,” “cash” or “gift.” The term bought and brought goods was not included in the search terms, since this term is rarely used in scientific literature. All three of the keyword themes were initially searched separately in PubMed, CINAHL, Web of Science, Worldwide Political Science Abstracts and Applied Social Sciences Index and Abstracts. A combined search of the separate searches was then completed with the Boolean operator AND. The following specific journals that typically provide information on informal patient payments or healthcare governance were manually searched: the Journal of Public Health, The Slavonic and East European Review, Health Policy and the International Journal of Healthcare, Insurance and Equity. Searches were also completed at the websites of the World Health Organization (WHO), the Regional Cooperation Council (RCC), the United Nations Office of Drug and Crime (UNODC), the UN Development Programme (UNDP), the EU and all the Western Balkan Institutes of Public Health. The public health institutes’ websites in Kosovo, Montenegro, and Bosnia and Herzegovina were in the local language and not in English, and it was therefore not possible to complete searches at these websites. Publication bias^35^ is therefore a risk because there may be valuable information at these websites that remains undiscovered.

### Selection of Studies

A study was included based on the following criteria: the study relates to one or more Western Balkan countries; it is a primary study that addresses the issue of informal patient payments or bought an brought goods (voluntary or forced gifts, unofficial cash payments or illegitimate goods bought and brought by patients to hospitals or clinics); it is written in English; and it uses data from the 2002–2015 period. Exclusion was based on duplication or a failure to fulfill the inclusion criteria. Papers addressing suggestions to tackle informal patient payments were identified when extracting the data and were therefore not an inclusion criterion. A total of 3319 studies were initially identified; of these, 3068 remained for title screening after 251 duplicates were excluded in EndNote (Figure). The title screening excluded 2870 studies based on the inclusion criteria, including 198 papers for abstract screening. Of these, 127 were excluded based on the inclusion criteria, and 71 were included after the articles were read in full-text. An assessment against the inclusion criteria was carried out, which resulted in the rejection of 38 studies that were not relevant to the research question. The 33 remaining papers were assessed for their quality using tools from the National Institute for Health and Care Excellence (NICE).^36^ Different tools were used for qualitative, quantitative and intervention studies. Nine studies were rejected due to poor quality, which left 24 studies with good (++) to moderate (+) quality for the final synthesis.

**Figure:**
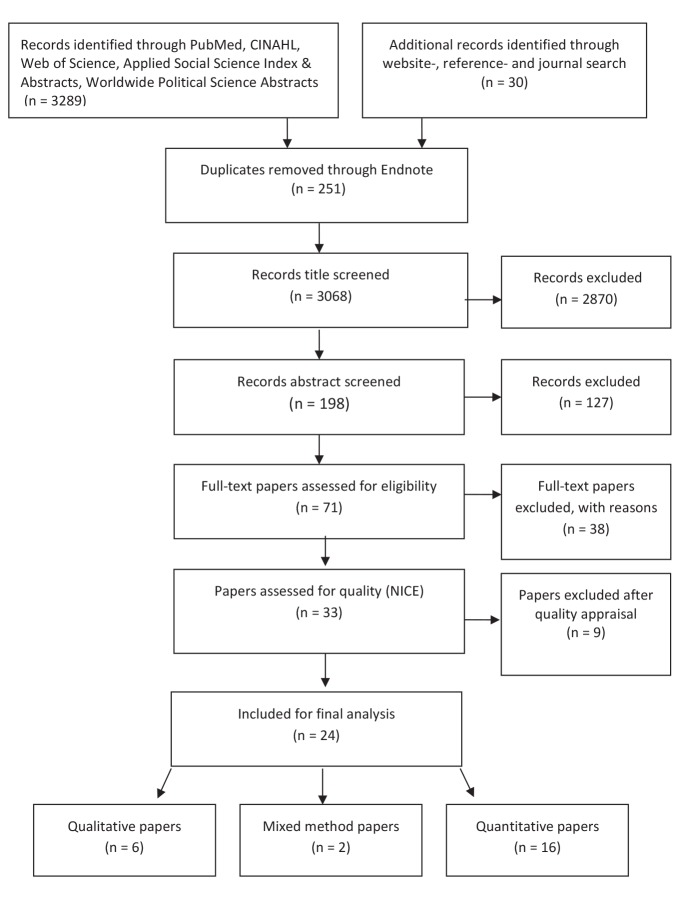
PRISMA Diagram of Included and Excluded Literature. Abbreviation: NICE, National Institute for Health and Care Excellence.

### Data Extraction and Synthesis Methods

The data that were relevant to the research question from the 24 included papers were extracted from the texts and saved in an Excel sheet. Data extraction was repeatedly complemented until it was found to be satisfactory.

The synthesis of evidence was performed based on the thematic synthesis method developed by Thomas and Harden.^37^ The authors describe the process of a thematic analysis comprising three stages: free coding of the primary studies, organizing the codes in descriptive themes and developing analytical themes to integrate the findings of multiple studies. The results were also explained in a narrative manner. Coding the text (both narratives and numbers) and dividing it into preliminary descriptive themes was completed in Excel. The preliminary themes were then operationalized into analytical themes that directly addressed the research questions of the review. These analytical themes extended beyond the preliminary findings and generated additional understandings or hypotheses.^37^ The analytical themes were identified to answer the posited research questions through the judgments and insights of the researchers with regard to the results. The preliminary descriptive themes were outlined on paper (not in Excel) to facilitate comprehension of the data. Next, lacking evidence was considered, and remedies as well as respondents’ perceptions and attitudes towards informal patient payments and bought and brought goods were described. After describing the studies, these analytical themes will structure the findings section.

### Grading Evidence

The review findings were assessed for confidence based on the Confidence of the Evidence from Reviews of Qualitative Research (CERQual).^38^ Although it is a qualitative tool, the researchers found that it functioned well in grading qualitative, quantitative and mixed method studies because a narrative approach was taken to synthesize findings and draw conclusions from the included studies. The grading consisted of four components: methodological limitations, relevance, coherence and adequacy.^38^ These four components were operationalized into an overall assessment of the levels of confidence in evidence: high, moderate, low or very low. Because the CERQual tool was used before recognizing the analytical themes of the thematic synthesis method, the assessment is based on the preliminary descriptive themes.

In terms of the methodological limitations of the three types of studies (quantitative, qualitative or mixed methods), these were graded minor, moderate or substantial concerns due to the three levels of assessment (-/+/++) in the NICE methodological assessment tool. Studies were primarily graded as either minor or moderate. Studies with substantial methodological limitations, such as poor representation of the source population or lacking reporting of evidence, were excluded. Methodological considerations within the reviews mainly involved minor concerns regarding validity or the inclusion of too few participants in the studies (mostly qualitative studies), whereas saturation of data may not have been fulfilled. Findings are however reliable and policy makers and users of the review may place reasonable emphasis on these findings.

## Results

### General Description of Studies

The 24 included publications consisted of 17 articles in scientific journals, four reports, two master’s theses and one working paper, all of which were published between 2002 and 2015. The fieldwork that is reported in these publications had taken place between 2002 and 2014. The sampling area varied from districts, municipalities, cities and towns to general populations in a single country and in multiple countries. Moreover, informal patient payments – and to some extent bought and brought goods in healthcare – were mostly examined from the perspective of the general public because several of the studies included data from surveys, particularly surveys that measured living standards. The sample sizes also varied: nine of the 24 studies included samples that were larger than 10 000; in 10 of the studies, the sample sizes were between 100 and 8000; and five of the studies included fewer than 99 respondents. Respondents were not the only source of data, as two of the studies supplemented their data with a literature review, including governmental and non-governmental literature such as government decisions, legislation, books and journals.

Seven of the studies were cross-national, whereas the remaining 17 studies were focused on a single country. Research on informal patient payments was found in all the Western Balkan countries, including the following: Albania (14), Serbia (9), Kosovo (7), FYROM (6), Bosnia and Herzegovina (5), Croatia (5) and Montenegro (4). However, research on bought and brought goods was conducted only in Serbia. Other studies mention these practices with concrete names such as ‘paying for drugs’ or ‘paying for an epidural analgesia’ or ‘paying for the provision of a bed.’ The data on bought and brought goods were thin or unclear in Albania, Bosnia and Herzegovina, Kosovo and FYROM (Table).

**Table:** Summary of the Findings

**No/Ref**	**Country**	**Sample Size**	**Focus and Aim**	**Setting**	**Type**	**Qual.Score**	**Main Findings**	**Described Effects**	**Perceptions on Interventions**
(1) Arsenijevic et al, 2015	SER	17 375	Evidence on bought and brought goods, formal and informal patient payments in the public healthcare	General pop. single country	Quant	++	Only about 5% of healthcare users report informal patient payments, whereas around 60% report paying bought and brought goods.	Payments for bought and brought goods present the highest share in the annual household consumption per household member. The burden is minor for informal patient payments. Rural residents, poorer and non-married report more payments for bought and brought goods. Young and more educated report more informal patient payments.	
(2) Arsenijevic et al, 2014a	SER	45 127	OOP payments for in- and outpatient care by exempted groups	General pop. single country	Quant	+	All population groups report informal patient payments in 2002, 2003, and 2007. OOP payments (formal, informal and bought and brought goods) in exempt groups (elderly, children, unemployed, disabled, poor) are less frequently reported for outpatient care than for inpatient services, except for the year 2007.	Elderly and patients with low income pay more for pharmaceuticals, disposable materials and orthopedic devices brought by patients to the hospital than other population group.	
(3) Arsenijevic et al, 2014b	SER	657	Quality and access indicators and patient payments for maternity care in Serbia	Maternity patients, District/municipality	Mix	+ Quant ++ Qual	21% paid informal patient payments for maternity services. Quasi-formal payments (payments organized by the facilities in the absence of government regulations) are charged for standard services that should be provided for free. Recipients of informal patient payments are obstetricians, anesthesiologists, midwives or nurses. Informal patient payments are given to secure obstetricians’ presence during childbirth, better quality of care, and the timely application of epidural analgesia. The highest amount of reported informal patient payment is 500 Euro.	Almost 90% of bribers continue to experience inconveniences during stay in hospital. Some did not report bribes but indicated having special connections to ensure better treatment and care. Women with connections report fewer inconveniences than those who have paid informally.	
(4) Avdyli, 2010	KOS	39	Perception of how informal patient payments affect the quality of and access to healthcare	Patient and providers, District/municip.	Qual	+	Informal patient payments are accepted due to poor healthcare system governance. They are necessary to receive care or better quality care or are given in gratitude or to maintain a positive relationship. Some providers do not receive informal patient payments for fear of getting caught, for ethical reasons or because they choose to work in public and private clinics to earn more money.	Informal patient payments are used to support further education of doctors, increase living standards, increase professional growth, maintain a professional level of care, act as counter-favors between doctor and patient, and increase happiness among the staff.	Providers suggest a performance-based system of payments or legalization of informal patient payments
(5) Bredenkamp, 2011	ALB BH KOS MON SER	49 848	The effects of health-related expenditures on household welfare	General pop. multiple country	Quant	+	Informal patient payments are substantial in all countries, but particularly high in Albania. They represent a large share of total health expenditure, often among the poor. In Albania, the poorest households pay higher health expenditures in informal patient payments than the richer quintiles. In Serbia, the rich pay a slightly greater share of health expenditure in informal patient payments than poorer. In Kosovo, expenditure shares are almost the same across quintiles.		
(6) Budak and Rajh, 2012	CRO	3005	Underreporting of corruption	General pop. single country	Quant	+	92% of the 14% within the study population with corruption experiences claimed gifts to providers, mostly voluntary. Few reported corruption experiences formally as they gained benefits from the bribe, believed nothing useful would come from reporting, or had given a gratitude gift. People reporting corruption was perceived as likely to regret it.		
(7) Vian and Burak, 2006	ALB	222	Intentions, past behaviours, attitudes and beliefs about informal patient payments in government health facilities	General pop. Cities/towns	Quant	++	No significant demographic difference was found among people intending to make informal patient payment vs. those that did not. Intenders were more positive in attitude and consequences of informal patient payments than non-intenders. The practice was believed unethical and illegal but did not influence people’s intention. Informal patient payments were beneficial and were necessary to obtain quicker and better care or medical attention. Intenders are more likely to believe they will get faster and better quality care than non-intenders, but they also believe that they must pay to receive any care at all.	People not intending to pay informally more often report having connections with medical personnel.	
(8) Vian et al, 2006	ALB	131	Help health planners to understand informal patient payments in government health facilities	General pop. and providers, Districts/municipalities	Qual	+	Factors promoting informal patient payments are perceived low salaries of health staff, a belief that good health is worth any price, to obtain care or better service, security or fear of being denied treatment, lack of social connections, and the custom of gratitude gifts. For one person, payments for healthcare are reported up to 50 000 Albanian LEK (approx. 380 Euro) per transaction.	Informal patient payments create uncertainties and anxiety during the care-seeking process, harm providers’ professional reputation, induce unnecessary medical interventions, and create discontinuity of care or better patient-provider relationships.	
(9) Colombini et al, 2012	ALB MAC	58	Access of Romani in S-E Europe to sexual and reproductive health services	Roma people, Cities/towns	Qual	++	Romani’s report paying up to 11 000 FYR Macedonian MKD (approx. 177 Euro) per transaction for one person, while also paying for care despite having health insurance.		
(10) Tomini and Maarse, 2011	ALB	17 302	How patients’ characteristics influence informal patient payments for in- and outpatient care	General pop. single country	Quant	+	Informal patient payments for healthcare services are widespread in in- and outpatient care and are dependent on certain characteristics of patients, including age, area of residence, education, health status, and health insurance. These payments are less dependent on income. Payments are higher in inpatient care than in outpatient care. Patients paid either voluntarily or because they were requested to.	Patients with chronic illnesses, the lower educated, or rural residents were more likely requested to pay informally to medical staff.	
(11) EBRD, 2011	ALB BH CRO KOS MAC MON SER	Not clear	How transition is affecting the lives of people in CEE, SEE, the Baltics, CIS and Mongolia	General pop. multiple country	Quant	++	Informal patient payments to public health officials were made mostly because they were asked to or it was necessary. To a lesser extent, gratitude gifts to doctors were given. The highest reported incidents of informal patient payments were reported in Albania with 39% of respondents reporting making such payments.	Lacking drugs in healthcare reduces the satisfaction in services. The likelihood that patients are asked to or voluntarily bring pharmaceuticals increases.	
(12) UNDOC, 2011	ALB BH, CRO KOS MAC MON SER	28 066 people	Gain knowledge about and learn from people’s experiences with corruption in the Western Balkans	General pop. multiple country	Quant	++	On average, one in six citizens of the region experiences bribery with a public official annually, and 12% of citizens pay at least one bribe. Corruption in the general public sector is mostly in rural areas and cash payments account for two-thirds of all bribes. The average bribe across the region is 257 EUR and as high as 1000 Euro. Bribes are paid in response to a direct or indirect request, offered voluntarily, or to receive better treatment at the doctor.	The average number of bribes paid is higher among lower income groups than wealthier citizens. No social group is exempt from bribery.	
(13) Grødeland, 2013	SER KOS MAC	1900	Public perception of corruption, types of corruption, institutions responsible for anti-corruption reform and anti-corruption efforts	General pop. multiple country	Mix	+ Quan ++ Qual	Small gifts or amounts of cash are reported. Payments were perceived as corruption, due to socialism or transition, to be necessary or a custom (gratitude), to get access or quicker access to healthcare services, for physical comfort, or to supplement salary of providers. Respondents were asked or volunteered to bring or buy goods for healthcare services supposed to be provided for free. Informal patient payments were max. 50 EUR but bought and brought goods were more than 1000 EUR per transaction for one person.	Informal patient payments were avoided in informal networks (People who are able and willing to help each other, providing information access to other people or assistance) between doctors and patients and payments were typically in vulnerable groups.	Study participants suggest to change mentality; use organized religion to indicate morality and reduce informal patient payments; prosecuting and sentencing corrupt politicians, government officials and others engaging in corruption; strengthen powers of institutions fighting corruption; and introducing new anti-corruption legislation. Measures would be successful with political will, stricter laws and law enforcement, higher living standards or use of force.
(14) Hotchkiss et al, 2005	ALB	2000 HH	Understand the magnitude and distribution of OOP payments and identify factors why and how much people pay	General pop. Cities/towns and districts/municipalities	Quant	++	45% of those requiring hospitalization reported extra fees for provider services and 61% report making gift payments. 44% of those using outpatient services for acute health problems reported paying (unofficial) consultation fees and 25% providing gifts. Gifts were mostly voluntary.	Cost of services was a reason for not using healthcare. Of patients with acute health problems, the poor were more likely to pay for consultations, but were less likely to make gift payments than better off clients. Clients of polyclinics and hospitals paid OOP payments more frequently than clients of PHCs and ambulances. Rural residents paid more frequently for consultations. Socio-economic status did not affect paying informally.	
(15) Janevic et al, 2011	MAC SER	90	Develop a conceptual framework showing how levels of racism affects access to maternal healthcare in Romani women	Roma women, gynecologists, NGOs and state institutions, cities	Qual	++	Informal patient payments were cited in all focus groups and social connections were believed to give access to better quality service. The ability to make informal patient payments or contribute gifts influenced the quality of service and level of negligent behavior toward Romani women.	The perceived or actual need to pay bribes reduced the likelihood of seeking maternal healthcare.	
(16) Krupic et al, 2015	BH	21	How immigrants and refugees experience different institutions and the health system	Refugees, cities/ towns	Qual	++	Informal patient payments were given to receive access to care or to receive better care.		
(17) Uka, 2013	KOS	29	Perceptions of informal patient payments	Patients and providers	Qual	+	IP are a result of culture and custom rather than socio-economic conditions. Payments vary by level of healthcare, department, urgency of treatment, and patients’ attitude towards informal patient payments. Seventy percent of patients had offered informal patient payments for themselves or for a family member. Most doctors denied receiving IP. IP was most prevalent in surgery, gynecology and obstetrics. Patients pay to receive better care and subsidized drugs, to skip the queue, for a preferable doctor, and/or to express gratitude.		
(18) Radin, 2013	CRO	2300	Assess the relationship between corruption and trust in public healthcare	General pop. single country	Quant	++		Corruption has negative effects on trust in public healthcare in the 2007 survey but not in the 2009 survey. Perceptions of and experience with corruption is negatively correlated with choice of public healthcare facilities, suggesting lower trust in public care providers. Patients are more willing to provide drugs to healthcare when there are shortages of medicine.	
(19) RCC, 2015	ALB, BH, CRO, KOS, MAC, MON, SER	7000	Examine public opinion on various topics covered in the five pillars of the SEE 2020 Strategy	General pop. multiple country	Quant	++	Respondents believe that giving or taking bribes is widespread in people working in the public health sector in Croatia (17%), Serbia (38%), Bosnia and Herzegovina (28%), FYROM (23%), Kosovo (13%), Albania (24%) and Montenegro (30%).		
(20) Tomini et al, 2013	ALB	10 840 people	Analyse how much OOP health spending impoverishes households	General pop. single country	Quant	+	There is lack of clarity between formal and informal patient payments in Albania. The amount of informal patient payments per capita has increased substantially over the years, from 220 (1,6 Euro) ALL in 2002 to 384 (2,8 Euro) ALL in 2008 on average per transaction for one person.	The amount paid informally by the lowest quintile increased almost five times over the years, while there was a more moderate increase in the higher quintiles over the same period. On average, the poorest households had relatively less budget than the rich but faced higher OOP payments.	
(21) Tomini, 2007	ALB	7973 People and 1782 HH	Identify determinants of informal patient payments in healthcare	General pop. single country	Quant	++	There are differences between the determinants of informal patient payments in inpatient and outpatient care. For example there are reasons to believe that if services of inpatient sector are purchased by health insurance we may observe less informal patient payments.	If services of the inpatient sector are purchased by health insurance there may be less informal patient payments (at least for some categories). Other determinants were higher level of incomes, positive health rating, lower level of education, and services offered by public providers.	
(22) Tomini et al, 2012a	ALB	7238 HH	Intra-household differences in spending on informal patient payments	General pop. single country	Quant	++	There are no significant differences between household members’ incidence of informal patient payments, but there are more differences in the amount paid informally. Thus, households strategically favor individuals with higher earning potential (human capital).		
(23) Tomini et al, 2012b	ALB	7238 HH	Measure the amounts of informal patient payments	General pop. single country	Quant	+	Medical staff has less information on patients’ maximum willingness to pay informally than patients have on medical staff’s minimum expected amount.	Informal patient payments are characterized by rural residents paying lower amounts; household size is negatively related to the amount paid; older individuals pay smaller amounts in inpatient care; patients are either asked or expected to pay informally; higher educated or patients married or living together pay higher amounts informally; and health insurance decreases the amount paid informally.	
(24) Tomini and Groot, 2013	ALB	10 839 HH	Explore the demand side of informal patient payments in in- and outpatient care	General pop. single country	Quant	++		Incidents of informal patient payments are highest among rural residents; lower educated patients; those with difficulties paying for healthcare; patients with less information on the amount required, patients with fewer social connections. Having national health insurance lowers the probability of paying informally both for outpatient and inpatient care.	

Abbreviations: HH, households; OOP, out-of-pocket; PHC, primary healthcare; NGOs, non-governmental organizations.

### Definitions and Measures of Informal Patient Payments

Most of the included studies defined in their papers informal patient payments as being either voluntary or forced payments or gifts to healthcare professionals. Other studies (#16,#18,#19) inexplicitly described informal patient payments as unofficial fees to health staff and used terms such as ‘corruption to healthcare staff’ or ‘bribes to public health officials.’ Several studies (#1,#2,#4,#6,#7,#8,#10,#11,#12,#13,#14,#15,#21) examined both gifts and cash, dividing their data into these two categories. Other studies (#5,#9,#17,#20,#22,#23,#24) combined voluntary and forced gifts and cash payments and used the general term ‘informal payments’ to describe and analyse their data. Only two studies (#1,#2) explicitly studied bought and brought goods.

### What the Data Reveal About Informal Patient Payments and Bought and Brought Goods

The reviewed papers revealed a variety of descriptive data on the phenomenon of informal patient payments. However, limited data were found regarding the specific issue of bought and brought goods. In the remainder of this study, bought and brought goods will be referred to only when data are available. The numbers in brackets in the text below refer to the number of included papers. The main findings (what is known, the perceived effects on healthcare and the perceived remedies for the practice of informal patient payments) are collected in the Table.

The reviewed studies will be referred to in brackets, marking their number from #1 to #24. Complete list of included studies may be found in the Appendix 1, separate from the reference list.

#### Incidents of Payments for Healthcare in the Western Balkans

The results showed that ‘incidents of informal patient payments’ as an overall aim were studied most often. Few studies divided these payments into cash payments and gifts, thus providing only a limited picture of these payments. Quantitative studies and some qualitative studies reported the incidents in percentages, whereas other qualitative studies identified narratives. Quantitative findings on incidents were divided into percentages of respondents in the studies who reported informal patient payments (gifts, bought and brought goods or informal patient payments in general) or into percentages of annual household and per capita healthcare expenditures measured in terms of informal patient payments. In this regard, the respondents in the included studies reported making informal patient payments from 4% up to 91% of the time, which indicates a great diversity in the reporting. Out of the 24 studies, 15 identified incidents of informal patient payments in all sectors of healthcare, while eight divided their data into outpatient and inpatient care. In the 15 studies, the incidents of informal patient payments most commonly took place in outpatient care. Two studies (#20,#5) in the review describe overall per capita expenditures on formal payments, including bought and brought goods. These data are not included in this study because it is unclear whether the described goods are legitimate or illegitimate or both.

The limited data that are available on bought and brought goods is illustrative in that these goods were reported in the studies in less than 1% of the study population (in Serbia and Kosovo) and as high as 90% of the study population (in Albania). These data are uncertain because the term bought and brought goods is not specifically used to describe all of these transactions. However, researchers indicate that such payments are illegitimate and that healthcare is supposed to be provided to patients for free. In addition, some of the data were not included for the following reason, eg, one study (#12) reports “other goods” to be paid by patients, although it is uncertain what is included in that category. Nonetheless, two Serbian studies (#1,#2) examine the illegitimacy of bought and brought goods in inpatient and outpatient care, and they report as high as two-thirds of their study population bringing or buying these goods in healthcare although healthcare services are supposed to be provided for free. Further, respondents in two of the qualitative studies (#4,#13) reported that they had to bring goods to the hospital when they were being treated.

Informal patient payments were widely reported in Albania. Within the entire healthcare system, the Albanian respondents reported paying informally between 19% and 91% (#7,#11,#12) of the time. The corresponding figures in Serbia were between 7% and 23% (#3,#11,#12), 7% and 70% in Kosovo (#5,#12,#17), up to 17% in Croatia (#11,12), between 5% and 22% in Bosnia and Herzegovina (#11,#12), between 8% and 13% in Montenegro (#11,#12) and between 4% and 13% in FYROM (#11,#12). The respondents in seven studies (#4,#8,#9,#13,#15,#16,#17) qualitatively explained their experiences with informal patient payments, eg, that they had been asked or had volunteered to make informal patient payments. The Balkan Barometer Study (#19) found that up to nearly 40% of the respondents in each Western Balkan country believed that practices of giving or taking bribes or abusing positions of power for personal gain are widespread among public health officials.

#### Paying Informally in Inpatient and Outpatient Care

Some studies typically divide their data on informal patient payments into inpatient and outpatient care. In Albania’s inpatient care, informal patient payments were reported by 60% of the respondents (#24). In Serbia the corresponding number is 19% (#1). Moreover, seven qualitative studies (#4,#8,#9,#13,#15,#16,#17) identify respondents as having to pay informally to receive (better) healthcare at hospitals. With regard to Albanian outpatient care, 28% (#24) of the respondents report making an informal patient payment, whereas in Serbia it is only approximately 2% (#1). Dividing these payments into gifts (ie, food, drinks, jewelry) in inpatient and outpatient care, the gifts are reported in up to 11% of the respondents in Serbian outpatient care (#1) and in 28% of Albanian outpatient care (#21). In inpatient care, the largest percentage of respondents who report gift giving (ie, food, drinks, jewelry) is 46% in Serbia (#2) and 61% in Albania (#14). The incidents of cash payments in healthcare are poorly reported in the quantitative studies. In one Serbian study (#1), less than 1% of the respondents reported informally paying cash in outpatient care and up to 3% (#2) in inpatient care. A survey study of all Western Balkan countries (#12) examined the forms of payments to all public officials and found that there is substantial difference between countries in terms of either paying in cash or providing food and drinks or other goods. In this study, cash nevertheless appears to be the most common form of bribery in two-thirds of all bribery cases.

#### The Forms and Amounts Paid for Healthcare Services and Goods in the Western Balkans

Different methods were used to report the amounts of informal patient payments and bought and brought goods. In this study, only data that involve the paid amount (not percentages) are included because qualitative studies frequently provide narratives of these amounts.

Most commonly and particularly in Albania (#8,#10,#12,#14,#20,#22,#24), the respondents described informally paying up to 50 Euro in cash for healthcare services. Studies from Kosovo (#4), Serbia (#3) and one from all the Western Balkan countries (#12) found respondents paying as much as 500 Euro for healthcare services. The latter study (#12) also reported payments over 1000 Euro. Only two studies on Kosovo (#4), Serbia and FYROM (#13) found a description of the size of gifts that were given by patients. Small gifts were given mostly in gratitude, and large gifts, such as a hotel stays, were given in reciprocation for services such as free services at an automobile mechanic. The papers typically use the term “gifts” as an overall synonym for favors and gratuities.

A distinction should be made in the matter of paying informally for services or goods. Though the amounts that are paid for bought and brought goods are only described in two studies from Serbia, two from Albania and one from FYROM. The respondents from Serbia and Albania reported frequently paying up to 50 Euro for goods (ie, epidural or other medicine) (#2,#14,#20). Another study from Serbia (#3) found patients paying between 100 and 200 Euro for an epidural in maternity care, whereas in FYROM (#13) a respondent paid more than 1000 Euro for medicine that was supposed to be provided free of charge by the hospital.

The methods for measuring the amounts of informal patient payments varied across studies, and a comparison between studies is thus therefore not relevant because it merely provides an overall picture of the informal amounts that are paid for healthcare in the Western Balkans.

#### Drivers of Informal Patient Payments

##### Cultural Perceptions or Necessary Payments

The review revealed a picture of patients who wanted to express their gratitude to providers, whereas others felt that it was an obligation to pay for qualified service. In 14 studies, which cover all the Western Balkan countries, providers *asked* patients to pay for healthcare services. However, in nine studies, which also cover all the countries, the patients *volunteered* to pay cash or give gifts in either gratitude or out of fear of not receiving quality service. Most studies identify both patients and providers as initiators. Though, a great driver in all the countries to pay informally for healthcare was to express gratitude, which was mostly reported in Kosovo and Albania followed by Serbia, FYROM and Croatia (#4,#6,#8,#11,#12,#13,#17,#21). In six of the studies, the respondents also argued that informal patient payments were a custom (#4,#6,#8,#11,#13,#17) in that it was a gesture to thank the doctors. Conversely, five studies (#6,#7,#13,#15,#16) found that the respondents considered informal patient payments to be corruption. In the end, paying to receive access to or better quality healthcare services was found in all seven countries. Patients paying to gain access to health services were mostly reported in Serbia, Albania and FYROM (#3,#7,#8,#9,#11,#13,#14,#15), whereas paying to receive higher quality care was mostly reported in Serbia and Albania (#3,#7,8,#12,#14,#15). Another issue was being asked or volunteering to buy goods in exchange for healthcare services. For example, in one study (#3), a patient was asked to pay for an epidural analgesia in connection with childbirth. These studies were mostly from Serbia but these practices were also found, albeit to a lesser extent, in Albania, FYROM and Kosovo (#1,#2,#3,#13,#14,#17,#20).

It seems ambiguous as to whether patients pay to express gratitude or out of fear of receiving low quality care. However, a quantitative study of Albania (#23) found that the share of patients being requested or expected to pay informally was larger than those who paid voluntarily. Additionally, eight other studies in this review, from Serbia, Albania, FYROM, Kosovo, and Bosnia and Herzegovina, found that patients either gave a gift or gave cash to providers for their own psychological comfort (#3,#7,#8,#13,#14) or to supplement the doctors’ salary (#4,#8,#13,#16,#17). It is therefore difficult to argue that customary gifts and cash payments for gratitude to providers should remain in the system because in many cases they are required.

The review further identified informal patient payments that were given for transportation (#4,#14) or to receive treatment from a specific doctor (#4,#17). Moreover, in two studies from Kosovo and Albania (#4,#8), providers also described accepting payments to avoid insulting patients.

##### The Determinants of Paying Informally

Nearly all the studies – including studies from every country – described who is most likely to make informal patient payments. Only two studies examined who pays in bought and brought goods (#1,#2). The most common determinants that were studied included economic status, level of education, residency, age and belonging to a vulnerable group. Despite the comprehensive evidence, a review of the combined data set reveals that there is no definite indication of who in society pays the most. The contradictory data may be illustrated in that higher education was recognized in several studies (#10,#21,#22,#24) as a means of avoiding paying informally for care. It was however also found that higher educated patients were more likely to make informal patient payments (#1,#21) or to pay higher amounts in their informal patient payments (#1,#22,#23,#24). Dividing the data into countries, there is no apparent pattern. Often, only one study is conducted in an individual country, eg, in Serbia only one study (#1) identified that well-educated patients were more likely to make informal patient payments. Because the data are also thin in Kosovo, FYROM, Montenegro, Bosnia and Herzegovina, and Croatia, it is difficult to draw clear conclusions. Nevertheless evidence from seven studies from Albania (#8,#10,#12,#14,#21,#22,#24) indicates that rural residents pay more frequently than urban residents (#23,#24). Poorer groups in Albania were also found to pay more often than others (#5,#8,#20,#21,#24). However, richer populations have also been found to be more likely to pay informally (#8,#23,#24). In Serbia and FYROM, studies found vulnerable groups (ie, unemployed, poorer, elderly, Roma, immigrants) to be more likely to make informal patient payments (#2,#9,#13,#15), whereas in the other five countries there was either no or little evidence regarding this issue. The review further identified respondents making informal patient payments despite having health insurance (#4,#9,#14,#23), which indicates that insurance is not always helpful in reducing such payments.

#### The Effects of Informal Patient Payments

Most studies in the review described the effects that informal patient payments or bought and brought goods had on patients within the broader context of the healthcare system (Table). For example as discussed in the previous section, in some cases, vulnerable groups pay more often than others. These groups include people with poor social connections in healthcare, poorer populations and rural residents. According to the findings on bought and brought goods, a shortage of pharmaceuticals was found to reduce satisfaction in health services and to increase the likelihood that patients would either bring or be asked to bring such offerings when they undergo treatment (#11,#18). One Serbian study (#3) further found that patients continued to experience inconveniences or were dissatisfied with services after having paid informally, although patients who had social connections reported fewer such inconveniences. Another study (#8) found that patients expressed uncertainties and anxiety when seeking healthcare due to informal patient payments. In the same study, the patients believed that paying informally harmed the professional reputation of doctors, induced unnecessary medical interventions and led to discontinuity in care. However, informal patient payments were found to yield happier staff (#4) and better relationships between patients and providers (#4,#8). Doctors who benefit from informal patient payments also used such payments to further enhance their professional or private lives, eg, through education, travel, conferences or higher living standards (#4).

#### Perceptions Regarding Remedies to Fight Informal Patient Payments in Healthcare

Despite attempts to tackle informal patient payments and corruption in the Western Balkan countries, researchers believe that efforts appear to be limited.^39^ One Albanian study in the review (#20) found that informal patient payments have increased over the years despite anti-corruption strategies, which may be due (at least in part) to the quality and implementation of these types of payments (#13). It seems that a large majority of people in southeastern Europe have never been involved in the consultation process in which the government enacts legislation or engages in decision making (#19). Other studies in the review also address governments’ lack of will as an explanation for the preservation of informal patient payments. For example, a study on Serbian maternity care (#3) found a discrepancy between official and hospital guidelines, which led to quasi-formal charges to standard services. Several other studies (eg, #7,#8,#11,#12,#13,#16,#17) illustrated that reasons for respondents to make informal patient payments to healthcare staff was the poor adherence to law by both citizens and government officials and the lack of governmental effort to increase salaries and generally increase funding for healthcare. Therefore, informal patient payments were used by some doctors in Kosovo to attend courses or professional conferences and meetings (#4). Moreover, several studies (eg, #4,#8,#17) found that informal patient payments were mostly provided in hospitals to experienced doctors or within the most common areas of specialized medicine, eg, different surgery areas, radiology and maternity care. When respondents were asked about reporting illegal payments, they indicated that no actions were taken by the responsible institutions in response to such reporting and that the respondents additionally benefitted from these payments and therefore could not rationalize the reporting (#6,#12).

Exempting vulnerable groups from paying for healthcare services has been discussed in most studies, which indicates that policies on the issue appeared to have limited effect, as more than half the studies found vulnerable persons making informal patient payments or providing bought and brought goods (eg, #2,#5,#9,#14,#24).

Only three of the reviewed studies identified positive aspects of informal patient payments and perceptions of the initiatives that would reduce these payments. For example, in Kosovo (#4), some nurses shared such payments in a common box to be divided among them. To reduce informal patient payments, some providers in Kosovo suggested a performance-based system of payments or legalizing informal patient payments such that patients with more resources could receive better quality care (#4). On the other hand, some respondents from FYROM and Serbia believed that reporting should be improved and that the mentality of accepting informal patient payments should be changed (#13). To this end, some doctors would not accept informal patient payments due to ethical considerations (#4) or the fear of getting caught (#4,#13), whereas other doctors chose to work in both public and private clinics to earn more money (#4).

A few of the Albanian respondents believed that religious organizations might be used as a morality indicator to reduce informal patient payments, although this was only a minority of the respondents (#13). In the same study, the respondents indicated that the EU should exert pressure on the respective states to reduce corruption and that anti-corruption initiatives should be mainly addressed through the media, as it was most effective forum. In terms of effective initiatives, another study (#24) found a decrease in informal patient payments over the examined years, but it was unknown what measures were taken and why they may have worked. Only one reviewed study (#13) found measures that stand out in terms of respondents’ perceived effectiveness at reducing corruption: Prosecuting and sentencing corrupt high-ranking politicians, government officials and others who have engaged in corrupt behavior, strengthening the powers of legal and other institutions that fight corruption, and introducing new anti-corruption legislation. The respondents believed that these measures would only be successful with political will, stricter laws and law enforcement, higher living standards and/or the use of force.

## Discussion

In the review, we identified quantitative and qualitative studies in all the Western Balkan countries, combined with a variety of different respondents at the country-, city- and/or municipality-levels. The number of studies increased between 2002 and 2015, and most of the studies were conducted in recent years. In spite of the comprehensive evidence documenting incidents involving informal patient payments, it was clear that evidence was fragmented with cases of informal patient payments being reported on a wide scale across studies. In addition, evidence regarding which groups of society mostly pay for care was inconsistent and showed no obvious pattern. Findings of this nature may be attributed to the wide variety of areas and populations that are studied in the review, leading to an indecipherable pattern. On that account, similar findings were identified in a study from Nigeria, showing that no socio-economic group was exempt from paying informally. Neither urban nor rural residents were more exposed than the other.^40^ It may also illustrate a culture and a custom of gratuity and reciprocity in these countries. As the Western Balkan countries are processing to enter the EU, such potential customary practice warrants attention from decision makers. Measures primarily addressing healthcare access may thus not be successful if not tackling behavioral change as well. Stepurko and colleagues^41^ argue that a sensitive topic such as informal patient payments is difficult to estimate in terms of magnitude and real scope and, in particular, in terms of the frequency of occurrence. However, in this review, the value of informal patient payments to healthcare providers was more consistent, typically between 1-50 Euro, although some of the respondents also made informal patient payments as high as 1000 Euro for healthcare services. It is unknown what amounts of money are common in paying for care, as evidence on this matter is sparse. Further research in the area is required.

Both the quantitative and qualitative studies reported informal patient payments for inpatient care and to providers in common areas of specialized medicine, in particular. These findings are in line with the Living Standard Measurement Survey of the World Bank^42^ and studies from other Eastern European countries.^3,43-45^

Evidence of bought and brought goods was limited to only two studies that specifically examined this issue. Other studies included narratives from respondents on the issue, although much of the data were unclear because bought and brought goods were not specifically addressed or discussed. A limitation to our study is therefore the collection of data by others, which makes it difficult to assess whether the goods were legitimate or illegitimate. The data included on bought and brought goods were therefore included only in those cases in which researchers indicated their illegitimacy. Two of the reviewed studies (#11,#18) found that the absence of pharmaceuticals reduced the satisfaction with health services and increased the likelihood that patients would either bring or be asked to bring such payments themselves. In previous studies from the southern and eastern European area, the findings also illustrate contributions of medicine, food, bed linens or other materials that are supposed to be provided by the hospital during treatment.^46-48^ The practice of patient responsibility for bought and brought goods appears to have existed for many years, although this practice has not received the proper focus by decision makers and researchers in the Western Balkan countries. This review has provided greater knowledge of the existing literature available on bought and brought goods. Though further research should attempt to clarify the structure and cases of bringing and buying goods in the process of seeking healthcare in the Western Balkans because such practice appears to be unexplored. As with informal patient payments, bought and brought goods also appear to stem from a poorly governed healthcare system in which people apply a “do-it-yourself” approach. This type of approach is also referred to as alternative politics, where people are dissatisfied with the current government policy and consequently initiate their own actions. In this case, they bring and buy goods when they seek healthcare to ensure the quality of treatment.^49^ Lerberghe et al^50^ study the consequences of a poor working environment, such as underpaid staff and/or the lack of materials and medicine. These authors claim that corruption is a coping strategy for health providers to address unsatisfactory working or living conditions. Regulations and salary increases are not enough to tackle this problem, as professionals’ value systems and a pressure from users and peers is also necessary. A combination of these measures would most likely succeed in reducing corruption.^50^

The present review attempted to identify measures that might reduce informal patient payments. It was clear that few studies of the Western Balkan countries examined the reasons for effective or ineffective measures. However, some respondents in the review did contemplate measures that would reduce informal patient payments, and they found that legalizing informal patient payments and introducing performance-based systems of payments, strengthening reporting, changing mentalities, and involving the media, and/or including the EU or religious organizations in any prospective solution would help to reduce informal patient payments. Furthermore, respondents from one study in the review claimed that different judicial and regulatory measures would be successful. A successful strategy to reduce such payments appears to include both harder and softer measures, such as that suggested by Miller and Vian,^51^ which included legislative, judicial, political and financial restructuring and behavioral changes in the public. Together with Gaal and colleagues,^52^ these authors claim that a context-specific strategy is important, including an assessment of the motivators of patients and providers, the structure of the healthcare financing and delivery system, and the resources available. Especially considering behavioral change among the public was learned in Taiwan, where the cultural beliefs among patients and providers did not change when securing better access to healthcare and reducing informal payments.^53^ If not tackling informal patient payments and bought and brought goods properly, patients may choose to insure themselves outside the public healthcare system, obtaining private health insurances. In such cases, healthcare access will become unequal, since only wealthier population groups will have this opportunity. Gaal et al^52^ found that success stories involving efficient strategies to reduce informal patient payments are rare due to the complexity of the phenomenon. Nevertheless, they emphasize the importance of stable and adequate (public) funding for healthcare and comprehensive and well-sequenced policy instruments to tackle informal patient payments.^52^ It may therefore be questioned whether inadequate funding and financial cuts in public healthcare facilitates the informal payments and bought and brought goods in Western Balkan countries. To our knowledge, only limited evidence-based research is available on the effectiveness of initiatives to combat informal patient payments in the Western Balkan countries, and future research would benefit from such studies. The present study was a review of the perceived initiatives to tackle informal patient payments, excluding literature studies and potential policy or legislative analyses that could have highlighted this issue in more detail. However, no studies in the present review examined assessments of the effectiveness of measures from the perspectives of politicians or civil servants who work to implement informal patient payment reforms.

## Considerations Regarding Methodological Limitations and Confidence in Findings

Scoping review methodologies have been well described in the literature by several researchers, who have enhanced and clarified the methodology. However, unresolved matters persist, such as reaching a common and accepted definition and purpose(s) of scoping studies and development of the methodological rigor that is required to assess scoping review quality. Moreover, to point out unresolved matters or issues that needs further focus in a literature review, the completeness of data by other researchers is necessary. Data is also often collected with another focus in mind than that of the review, ie, not only including Western Balkan countries or focusing on informal patient payments. Consequently some knowledge might not have been reported in the studies of the present review. Furthermore, in the search strategy evidence addressing the effectiveness of studies was not considered inclusion criteria. Some studies may therefore have been missed. The final point of consulting stakeholders in the scoping review methodology could have been a valuable tool to validate the findings, though this stage was not feasible for this review. The present scoping review was nevertheless conducted in a comprehensive manner that included the CERQual assessment tool to assess the evidence. Because the tool looks at combined findings across studies, it was possible to assess whether the data were consistent, whether the research was studied in various contexts and how much data were available.

The findings of this review were mostly graded either of moderate confidence or of very low confidence. High confidence in evidence is generally rare because studies frequently reveal their own limitations. In our assessment, many of the findings lacked adequacy, coherence or had methodological  limitations.

The CERQual assessment recognized a moderate confidence in the evidence with regard to the findings on the incidents of informal patient payments, the attitudes towards informal patient payments and the types of informal patient payments (eg, payments made based on gratitude, low salaries, and limited access). The evidence was widely studied in all the Western Balkan countries, although some countries (such as Albania and Serbia) were studied more than others, making it difficult to generalize the results in some countries. Most of the studies were although consistent in terms of study population and setting by including the countries’ representable populations and relatively unbiased data. Nonetheless, the data outcomes on incidents were not highly consistent across the studies, although the data were reasonably consistent with regard to attitudes towards and types of informal patient payments. These data could therefore be assessed with confidence and provide a reasonable representation of incidents of informal patient payments and of Western Balkan peoples’ perceptions of informal patient payments and their reasons for giving or taking them.

Low evidentiary confidence was found regarding the magnitude of informal patient payments. Data on these amounts were somewhat consistent, although with some outliers. The findings were however mostly from Serbia and Albania. In addition, quantitative studies were in some terms biased, eg, lacking reporting or concerns about lacking precision of association between variables, whereas the qualitative studies offered rich data but with methodological limitations, eg, lacking reporting of analysis.

With regard to bought and brought goods, the types of such transactions (such as the lack of drugs, limited access), their magnitude and their incidence were rated very low, due to the lack of data on this subject, which influenced the consistency, adequacy and relevance of the evidence. Conclusions based on these data should therefore be handled with care, as it is uncertain whether the findings are substantially different from the actual phenomenon.

## Considerations Regarding Implications for Policy and Research

Systematic reviews are often valued by policy makers and other civil servants due to the processual and systematical consideration of evidence that gives rise to an iterative exchange between the data collected. Our research demonstrates that the need for research and policy makers to address the phenomenon of patients paying informally for healthcare services or goods in Western Balkan. To further explore this topic, researcher should focus on would be valuable to consider the perspectives of politicians and other civil servants working in the field of implementing reforms on informal patient payments. The effectiveness of measures taken to reduce this informal practice may then be clarified further. The evidence presented in this paper may provide credible awareness towards the need for behavioral change and policies to address informal payments and bought and brought goods in healthcare.

## Conclusion

In conducting this scoping review, it became apparent that countries such as Albania and Serbia were studied in more detail and more often than others. Both large-scale studies and smaller studies were included, yielding a broad picture of quantitative and qualitative data on the issue of concern, eg, that incidents of paying informally were not consistently reported across studies. Thus, there was no clear pattern of which patient groups that pay informally or whether payments are made out of gratitude or obligation. Both positive and negative effects were found, which suggests that vulnerable groups were more at risk of paying informally or that patients felt anxiety, uncertainty or dissatisfaction with services when they pay informally. The positive implications for healthcare services were happier staff, better patient-provider relationships and better living conditions. The phenomenon is complex because of the juxtaposition of gratitude payments versus obligatory payments. However, patients often felt the need to pay doctors who work in poor conditions with a lack of equipment and/or low salaries, to take their own actions towards poor government health policies. To clarify the policy measures taken towards the reduction of patients paying informally, it would be valuable to examine the topic of this paper from the perspective of policy makers and other civil servants in the field of healthcare. It was also revealed that despite the lack of evidence on bought and brought goods, these seemed to be even more common than informal patient payments. In terms of effective strategies to reduce corruption, the review found that success might be achieved by changing mentalities and by including different institutions, such as the media or the EU. Although informal patient payments have been studied and described in several settings, there remains a dearth of research on the effectiveness of strategies in a Western Balkan setting.

## Ethical issues

Not applicable.

## Competing interests

The authors declare they have no competing interests.

## Authors’ contributions

SBM, the main author of this article – collected and analysed the data and drafted the manuscript while LEK participated in writing the article. She was also the scientific and methodological advisor of the research project.

## Endnotes


[1] Is the % of publicly funded healthcare of the total health expenditure in a country. Total health expenditure is the sum of public and private health expenditure.


## Appendix 1

### Appendix 1. Index of Publications Included in the Review

#1. Arsenijevic J, Pavlova M, Groot W. Out-of-pocket payments for health care in Serbia. Health Policy. 2015;119(10):1366-1374. doi:10.1016/j.healthpol.2015.07.005
#2. Arsenijevic J, Pavlova M, Groot W. Out-of-pocket payments for public healthcare services by selected exempted groups in Serbia during the period of post-war healthcare reforms. Int J Health Plan Manag. 2014;29(4):373-398. doi:10.1002/hpm.2188
#3. Arsenijevic J, Pavlova M, Groot W. Shortcomings of maternity care in Serbia. Birth. 2014;41(1):14-25. doi:10.1111/birt.12096
#4. Avdyli H. Informal payments in Kosovo Hospital. Prizren: European Center for Peace and Development (ECPD) of the University for Peace and ECPD regional centre for International postgraduate studies and development Research; 2010.
#5. Bredenkamp C, Mendola M, Gragnolati M. Catastrophic and impoverishing effects of health expenditure: New evidence from the Western Balkans. Health Policy Plan. 2011;26(4):349-356. doi:10.1093/heapol/czq070
#6. Budak J, Rajh E. Corruption survey in Croatia: Survey confidentiality and trust in institutions. Društvena istraživanjaČasopis za opća društvena pitanja 2012;2:291-313.
#7. Vian T, Burak LJ. Beliefs about informal payments in Albania. Health Policy Plan. 2006;21(5):392-401. doi:10.1093/heapol/czl022
#8. Vian T, Grybosk K, Sinoimeri Z, Hall R. Informal payments in government health facilities in Albania: Results of a qualitative study. Soc Sci Med. 2006;62(4):877-887. doi:10.1016/j.socscimed.2005.07.005
#9. Colombini M, Rechel B, Mayhew SH. Access of Roma to sexual and reproductive health services: Qualitative findings from Albania, Bulgaria and Macedonia. Glob Public Health. 2012;7(5):522-534. doi:10.1080/17441692.2011.641990
#10. Tomini S, Maarse H. How do patient characteristics influence informal payments for inpatient and outpatient health care in Albania: Results of logit and OLS models using Albanian LSMS 2005. BMC Public Health. 2011;11(1):375. doi:10.1186/1471-2458-11-375
#11. European Bank for Reconstruction and Development. Life in Transition Survey: After the Crisis. London: European Bank for Reconstruction and Development; 2011.
#12. UNODC. Corruption in the Western Balkans: Bribery as experienced by the population. United Nations Office of Drug and Crime, Vienna; 2011.
#13. Grødeland ÅB. Public perceptions of corruption and anticorruption reform in the Western Balkans. Slavonic East Eur Rev. 2013;91(3):535-598.
#14. Hotchkiss DR, Hutchinson PL, Malaj A, Berruti AA. Outof-pocket payments and utilization of health care services in Albania:Evidence from three districts. Health Policy. 2005;75(1):18-39. doi:10.1016/j.healthpol.2005.02.003
#15. Janevic T, Sripad P, Bradley E, Dimitrievska V. “There’s no kind of respect here” a qualitative study of racism and access to maternal health care among Romani women in the Balkans. Int J Equity Health. 2011;10:53. doi:10.1186/1475-9276-10-53
#16. Krupic F, Krupic R, Jasarevic M, Sadic S, Fatahi N. Being immigrant in their own country: experiences of Bosnians immigrants in contact with health care system in Bosnia and Herzegovina. Mater Socio Med. 2015;27(1):4-9. doi:10.5455/msm.2014.27.4-9
#17. Uka A. Understanding informal patient payments in Kosovo’s health Care system. Health policy Institute. Slovakia; 2013. http://www.hpi.sk/cdata/Publications/HPI_UKA_Understanding_informal_patient_payments_in_Kosovo.pdf. Accessed September 15, 2016.
#18. Radin D. Does corruption undermine trust in health care? Results from public opinion polls in Croatia. Soc Sci Med. 2013;98:46-53. doi:10.1016/j.socscimed.2013.08.033
#19. Regional Cooperation Council. Balkan Barometer 2015: Public Opinion Survey. Herzegovina, Bosnia: Regional Cooperation Council; 2015. http://www.rcc.int/seeds/files/RCC_BalkanBarometer2015_PublicOpinion_FIN_forWeb.pdf. Accessed September 15, 2016.
#20. Tomini SM, Packard TG, Tomini F. Catastrophic and impoverishing effects of out-of-pocket payments for health care in Albania: evidence from Albania living standards measurement surveys; 2002, 2005 and 2008. Health Policy Plan. 2013;28(4):419-428. doi:10.1093/heapol/czs073
#21. Tomini S. The Impact and the Determinants of Informal Payments in Health Care: The Case of Albania. Maastrict University, Graduate School of Governance; 2007. http://arnop.unimaas.nl/show.cgi?fid=11596. Accessed September 15, 2016.
#22. Tomini S, Groot W, Pavlova M. Informal payments and intrahousehold allocation of resources for health care in Albania. BMC Health Serv Res. 2012;12(1):17. doi:10.1186/1472-6963-12-17
#23. Tomini S, Groot W, Pavlova M. Paying informally in the Albanian health care sector: A two-tiered stochastic frontier model. Eur J Health Econ. 2012;13(6):777-788. doi:10.1007/s10198-011-0331-1
#24. Tomini SM, Groot W. Paying informally for public health care in Albania: Scarce resources or governance failure? Appl Econ. 2013;45(36):5119-5130. doi:10.1080/00036846.2013.818216
